# Surfactants decrease the toxicity of ZnO, TiO_2_ and Ni nanoparticles to *Daphnia magna*

**DOI:** 10.1007/s10646-015-1529-2

**Published:** 2015-09-26

**Authors:** Patryk Oleszczuk, Izabela Jośko, Ewa Skwarek

**Affiliations:** Department of Environmental Chemistry, Faculty of Chemistry, University of Maria Skłodowska-Curie, pl. M. Curie-Sklodowskiej 3, 20-031 Lublin, Poland; Institute of Plant Genetics, Breeding and Biotechnology, Faculty of Agrobioengineering, University of Life Sciences in Lublin, Akademicka 15, 20-069 Lublin, Poland; Department of Radiochemistry and Colloid Chemistry, Faculty of Chemistry, University of Maria Skłodowska-Curie, Lublin, Poland

**Keywords:** Nanoparticles, *Daphnia magna*, Surfactants, Toxicity

## Abstract

The objective of the study was the estimation of the effect of surfactants on the toxicity of ZnO, TiO_2_ and Ni nanoparticles (ENPs) towards *Daphnia magna*. The effect of hexadecyltrimethylammonium bromide (CTAB), triton X-100 (TX100) and 4-dodecylbenzenesulfonic acid (SDBS) was tested. The Daphtoxkit F™ test (conforming to OECD Guideline 202 and ISO 6341) was applied for the toxicity testing. Both the surfactants and the ENPs were toxic to *D. magna*. The addition of ENPs to a solution of the surfactants caused a significant reduction of toxicity of ENPs. The range of reduction of the toxicity of the ENPs depended on the kind of the ENPs and their concentration in the solution, and also on the kind of surfactant. For nano-ZnO the greatest reduction of toxicity was caused by CTAB, while for nano-TiO_2_ the largest drop of toxicity was observed after the addition of TX100. In the case of nano-Ni, the effect of the surfactants depended on its concentration. Most probably the reduction of toxicity of ENPs in the presence of the surfactants was related with the formation of ENPs aggregates that inhibited the availability of ENPs for *D. magna*.

## Introduction

In the near future engineered nanoparticles (ENPs) may become a new contaminant for the environment. This is indicated by the great increase of their production and application in various areas of life (Gottschalk et al. 2013). Both the production and the use of materials containing ENPs in their composition may, as indicated by recent research, lead to their release into environment (Gottschalk et al. 2011; Windler et al. 2012). Therefore it is extremely important to acquire detailed knowledge on the fate of ENPs in the environment, and especially on the factors that may have a significant effect on their mobility and, in particular, their toxicity towards various groups of organisms. Stability of ENPs in aqueous environments is a key factor controlling their transport and fate in aqueous environments (Sharma 2009; Lowry et al. 2012). In the aquatic environment ENPs interact among themselves and with other ENPs or larger particles. This process is determined by the properties of the ENPs as well as ENPs interactions with other compounds, both natural (e.g. natural organic matter, aquatic colloids) and anthropogenic (e.g. surfactants) (Lin et al. 2010). Aggregation reduces the overall specific surface area of ENPs and interfacial free energy and thus will limit the reactivity of ENPs (Saleh et al. 2008; Prathna et al. 2011; Lowry et al. 2012). The literature provides frequent indications of the effect of humic and fulvic acids on the aggregation of CNTs (Saleh et al. 2008). Another group that may affect ENPs solubility, mobility and dispersion are surfactants (Yang et al. 2010; Oleszczuk and Xing 2011). For example, nano-ZnO coated with the surfactant sodium dodecyl sulfate was stable in soil suspension for 14 days without changes in particle size distribution (Gimbert et al. 2007). Surfactants are often used to purify ENPs or as dispersants for application purposes (Bhushan 2010). Non-ionic and ionic surfactants are commonly used e.g. to coat nano-TiO_2_ to remain dispersed (i.e. stable) during the fabrication of paints and cosmetics (Tkachenko et al. 2006). In addition, in ecotoxicological studies surfactants are frequently used for the stabilization of ENPs, which may have a direct effect on toxicity. In view of the above information it is, therefore, extremely important to acquire knowledge on the effect of surfactants on the toxicity of ENPs. The few studies conducted so far have been concerned with plants and indicated an increase in the toxicity of ENPs in the presence of surfactants (Barrena et al. 2009; Stampoulis et al. 2009). Stampoulis et al. (2009) showed that sodium dodecyl sulfate confounded and, in most cases, amplified, the effects of ENPs on exposed *C. pepo* plants. Similarly, stabilizer coatings used to ensure the dispersibility and stability of Au, Ag, and Fe_3_O_4_ ENPs in water affected *Cucumis sativus* and *Lactuca sativa* (lettuce) seeds more than the ENPs alone (Barrena et al. 2009). Whereas, there is a lack of information on how surfactants may affect the toxicity of ENPs with relation to other organisms. Studies show that the toxicity of surfactants depends not only on their kind (cationic, anionic and non-ionic) but also on a number of other factors (e.g. their structure) (Ying 2006). Therefore, depending on what surfactant is used for the stabilization of ENPs one can expect diverse effects both on the part of the surfactants themselves, and on that of the surfactants and the ENPs.

The objective of the study presented here was the estimation of the toxicity of various ENPs—nano-ZnO, nano-TiO_2_ and nano-Ni in the presence of ionic (cationic—hexadecyltrimethylammonium bromide and anionic—4-dodecylbenzenesulfonic acid) and non-ionic (Triton X-100) surfactants towards *Daphnia magna*. It is estimated that among inorganic nanomaterials (apart from Ag) the highest production is characteristic of nano-ZnO and nano-TiO. ZnO and TiO_2_ NPs are widely used in the consumer products (sunscreen products, textiles, paints, coatings and antibacterial agents) which needs the detailed assessment of their potential toxicity to different organisms. The growing scale of production of NPs involves the risk of their release into the environment. Ni nanoparticles, on the other hand, are used in production catalysts, battery electrodes and diesel–fuel additives and also may released to environment. While for ZnO and TiO_2_ there are a lot of data on their toxicity, in the case of Ni NPs data on this subject are limited. Thus, it is important to evaluate the effect of the surfactants on both the common nanoparticles, such as ZnO and TiO_2_ but also less popular NPs, such as Ni NPs.

## Materials and methods

### Materials

Nanoparticles ZnO (nano-ZnO), TiO_2_ (nano-TiO_2_, mainly anatase form) and Ni (nano-Ni) were purchased from Sigma-Aldrich (USA). CAS numbers of used metal and metal oxide nanoparticles were: 1314-13-2 (nano-ZnO), 13463-67-7 (nano-TiO_2_), 7440-02-0 (nano-Ni). The ENPs (the purity was around 99.5 %) were used as powder. The primary particle size of ENPs was as follows: nano-ZnO < 100 nm; nano-TiO_2_ < 21 nm; nano-Ni < 100 nm. The size of ENPs was determined by transmission electron microscope (JEM-3010 TEM JEOL, Ltd., Japan). Surfactants (4-dodecylbenzenesulfonic acid—SDBS, hexadecyltrimethylammonium bromide—CTAB, triton X-100—TX-100) were purchased from Sigma-Aldrich (USA). All solutions were prepared using analytical grade reagents and HPLC grade water (POCH, Gliwice, Poland).

### Sample preparation

Samples of ENPs as well as surfactants were prepared in the ISO medium for Daphtoxkit F bioassay (5.75 mg/L KCl, 64.75 mg/L NaHCO_3_, 123.25 mg/L MgSO_4_ × 7H_2_O, 294.0 mg/L CaCl_2_ × 2H_2_O). In the each steps of experiment, the standard ISO medium without ENPs and surfactants was used as a control.

The first to be determined was the toxicity of solutions of the ENPs, and that of the surfactants. The toxicity of the ENPs was assayed within the range of concentrations from 0.05 to 1000 mg/L. Whereas, the toxicity of the surfactants was determined within the range of concentrations from 0.005 to 0.5 mg/L for SDBS and CTAB, and from 0.6 to 500 mg/L for TX100. The different ranges of surfactants concentration were tested because of their various toxicity towards *D. magna*.

For the purpose of determination of the effect of the surfactants on the toxicity of the ENPs such concentrations of the surfactants were chosen that caused immobility of the test organisms at the level of 10 %, i.e. for SDBS and CTAB-0.01 mg/L and for TX100-1 mg/L. The ENPs were added to surfactant solution, at the same range of concentrations at which their own toxicity was assayed (0.05–1000 mg/L). The solutions of ENPs and of the ENPs with surfactants were sonicated for 30–minute at temperature of 25 °C in an ultrasound bath (Polsonic, 250 W, 50 Hz) before application on the test plates.

### Bioassay

The Daphtoxkit F™ bioassay (Microbiotest, Ghent, Belgium) was used to estimate effect of surfactants on the toxicity of ENPs to crustacean *D. magna*. The whole procedure was carried out according to the user’s manual (Daphtoxkit 1996). The Daphtoxkit F test is performed in accordance with test procedures of OECD Guideline 202 and ISO 6341. The each test vessel contained 20 mL of the test solution and ten neonates (less than 24 h old).After 48 h the number of dead neonates was estimated. In order to check the correct execution of the test animals, the reference test was conducted with using the reference toxicant potassium dichromate (K_2_Cr_2_O_7_). The quality control test was successful.

### Sample characterization

The ISO medium with ENPs alone and ENPs with surfactants was characterized using dynamic light scattering (DLS) (Zetasizer 3000, Malvern), transmission electron microscopy (TEM) (Tecnai G2 T20 X-TWIN, FEI) and scanninig electron microscopy with energy dispersive spectrometry (SEM–EDS) (Quanta™ 3D FEG, FEI with EDAX SDD Apollo detector). For these analysis, the samples were prepared in following way: NPs at concentration of 100 mg/L were suspended in: (1) the ISO medium used in the test, (2) SDBS solution (0,01 mg/L), (3) CTAB solution (0,01 mg/L) and (4) TX-100 solution (1 mg/L) and pontificated for 30 min. The size of aggregates and zeta potential were measured by DLS technique **(**Zetasizer 3000, Malvern**).** The pH and O_2_ of samples was measured. The SEM–EDS analyses were conducted to observe nanoparticles on/inside *D. magna*. The SEM–EDS measurements were made with the high vacuum with accelerating voltage mode. Using the EDS maps it is possible to see Zn, Ti and Ni particles in/on *D. magna*. The EDS diagrams (below SEM–EDS maps) show spectrums, which present characteristics X-rays of sample atoms induced by electron beam (30 keV). The SEM–EDS maps (with 50-fold magnification) and were obtained for whole organism just after the exposure time. The EDS spectra confirm and correspond with the contribution of elements (Zn, Ti, Ni) in the whole sample area. In this particular case the organisms were taken from the solutions of NPs at concentration of 100 mg/L suspended in: (1) the ISO medium used in the test, (2) SDBS solution (0,01 mg/L), (3) CTAB solution (0,01 mg/L) and (4) TX-100 solution (1 mg/L).

### Data analysis

Effect concentrations (EC) were derived from full log-logistic (surfactants) or linear (ENPs) concentration effect curves. The reported EC_50_ values are the average of three independently replicates. The differences between toxicological data (EC50 for NPs only and NPs with surfactants) were evaluated using the Kruskal–Wallis test followed by post hoc Nemenyi test. Other differences between NPs and NPs with surfactants (particle size or zeta potential) were determined using a one-way analysis of variance (ANOVA) followed by Dunnett’s post hoc test.

## Results and discussion

### Effect of surfactants on *D. magna*

**Fig. 1:**
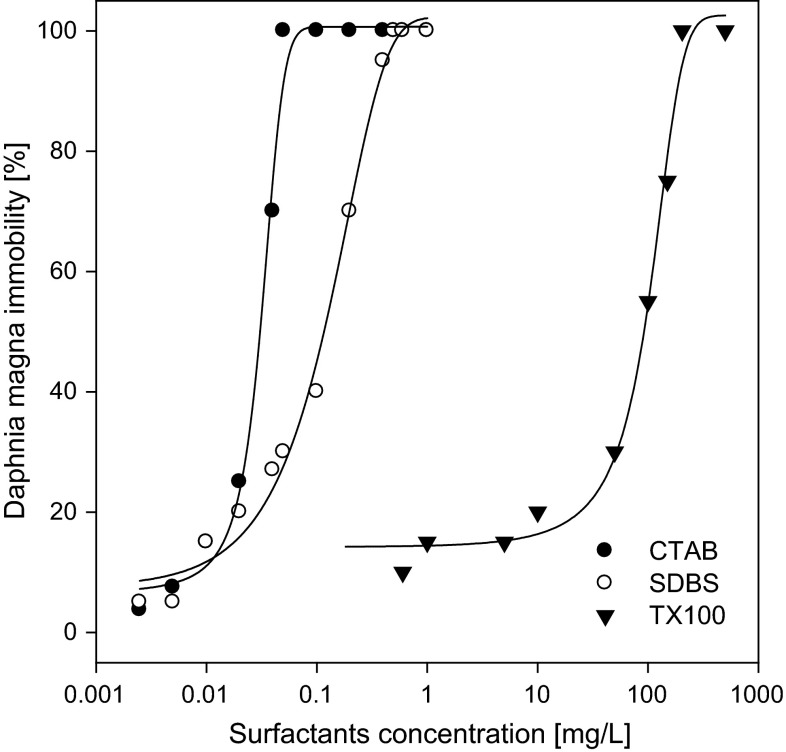
Effect of hexadecyltrimethylammonium bromide (CTAB), Triton X-100 (TX100) and 4-dodecylbenzenesulfonic acid (SDBS) on *Daphnia magna* immobility

Figure 1 presents the effect of the surfactants on the immobilisation of *D. magna*. The toxicity was varied with relation to the kind of surfactant applied. A gradual increase in the toxicity of the surfactants was observed with increase in their concentration. CTAB, already at the concentration of 0.05 mg/L, caused 100 % immobility of the test organisms. For SDBS, 100 % immobility was observed only after the concentration of that surfactant reached the level of 0.5 mg/L. The values of EC_50_ determined for CTAB and SDBS were 0.03 mg/L and 0.12 mg/L, respectively. The toxicity of TX100 towards *D. magna* was the lowest relative to the two other surfactants. The value of EC_50_ assayed for TX100 was at the level of 98.7 mg/L and it was higher by over two orders of magnitude compared to that for SDBS and by three relative to CTAB (Fig. 1).

The available literature data on the toxicity of surfactants are sparse. Surfactants input into environment through the discharge of sewage effluents into surface waters and application of sewage sludge on land. Therefore, estimation of the toxicity of surfactants is very important in the prediction of environmental hazard related with their presence in the environment. Lewis (1991) observed a chronic and sub-lethal toxicity of anionic, cationic and non-ionic surfactants with relation to aquatic organisms, that appeared at concentrations higher than 0.1 mg/L. Emmanuel et al. (2005) studied the toxicity of three surfactants (CTAB, TX100 and SDS) towards *D. magna* and observed that the toxicity of the surfactants was as follows: cationic surfactants > anionic surfactants > non-ionic surfactants. The same tendency was observed in the present study (Fig. 1). The highest toxicity towards *D. magna* was characteristic of CTAB, followed by SDBS. TX-100 was characterised by the lowest toxicity (Fig. 1). The values of EC_50_ calculated on the basis of the results were similar to those presented by Panouillères et al. (2007) and Emmanuel et al. (2005). The values of EC50 determined by those authors for *D. magna* were 0.087/0.024, 41.2/29.2 and 89.3/38.1 mg/L, respectively, for CTAB, SDBS and TX100. The sole difference observed in this study was a significantly higher toxicity of SDBS.

#### Behaviour of ENPs in ISO medium in presence of surfactants

**Fig. 2:**
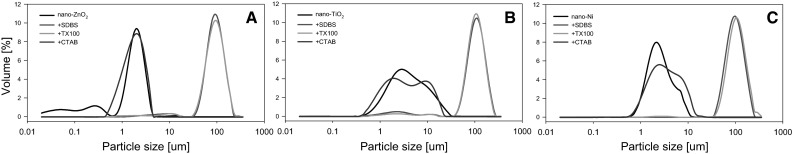
Influence of hexadecyltrimethylammonium bromide (CTAB), Triton X-100 (TX100) and 4-dodecylbenzenesulfonic acid (SDBS) on particles size of ENPs (at concentration of 100 mg/L) in ISO medium (dynamic light scattering (DLS) method). The concentration of surfactants in ISO medium: CTAB and SDBS—0.01 mg/L, TX 100—1 mg/L

**Fig. 3:**
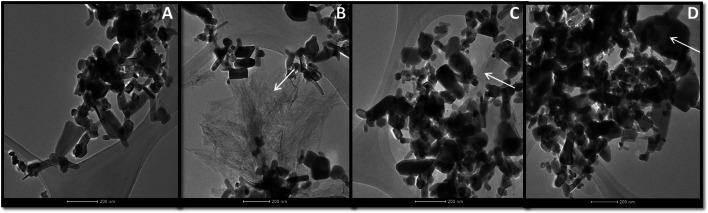
TEM pictures of nano-ZnO (**a**), nano-ZnO in CTAB solution (**b**), nano-ZnO in SDBS solution (**c**) and nano-ZnO in TX100 solution (**d**). The concentration of surfactants in ISO medium: CTAB and SDBS—0.01 mg/L, TX 100—1 mg/L. The concentration of nano-ZnO—100 mg/L. *Arrows shows* “the net” created by surfactants which connect nanoparticles

Figure 2 presents the size of nanoparticle aggregates and individual ENPs in the presence of surfactants in ISO medium. All of the ENPs studied appeared in the solution in the form of aggregates with sizes from about 1 to 10 μm (Fig. 2). The addition of ENPs to solution containing surfactants TX100 and SDBS caused a significant increase in the size of all of the ENPs studied. Whereas, ENPs addition to a CTAB solution had no significant effect on the size of nano-ZnO, nano-TiO_2_ and nano-Ni (Fig. 2). In the solution one could observe distinct connections between ENPs aggregates, in the form of bridges (web) formed by CTAB or SDBS (Fig. 3b, c). In the case of TX100, aggregates and individual particles were”coated” by the surfactant (Fig. 3d).

**Fig. 4:**
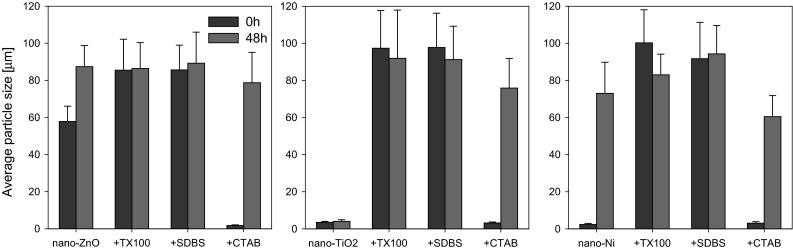
Average particles size of ENPs(at concentration of 100 mg/L) measured with DLS techniquealone and in surfactants solutionat (the concentration of surfactants in ISO medium: CTAB and SDBS—0.01 mg/L, TX 100—1 mg/L) at the beginning of the experiment and after 48 h

SDBS and TX-100 induced the increase of ENP-surfactant complex sizes from the beginning of the experiment. CTAB also caused an increase in complex size but only after 48 h (Fig. 4). Moreover, for nano-ZnO, CTAB significantly reduced the ENP size at the beginning of the experiment. After 48 h of the experiment, in the solution containing nano-ZnO or nano-Ni without surfactants their significant aggregation was observed (Fig. 4). Whereas, time did not have any significant effect on the mean size of aggregates of nano-TiO_2_. Also in the solutions containing TX100 and SDBS and all of the ENPs tested no significant increase of aggregate size was noted. Only in the case of CTAB after 48 h a significant increase of aggregate size was noted for all of ENPs tested. The mean size of the aggregates, however, was still smaller than in the case of the remaining surfactants and the ENPs.

**Fig. 5:**
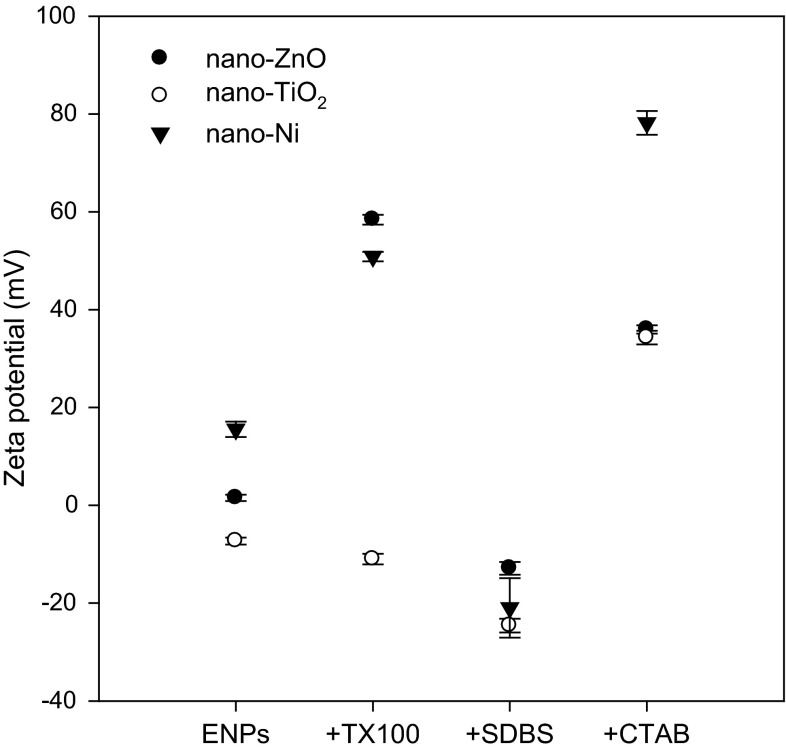
Zeta potential of ENPs (DLS technique) alone and ENPs (at concentration of 100 mg/L) in surfactant’s solution (the concentration of surfactants in ISO medium: CTAB and SDBS—0.01 mg/L, TX 100—1 mg/L)

ENPs differed from one another in the values of the zeta potential (Fig. 5). In the system under study, nano-ZnO and nano-Ni were characterised by a positive surface charge, while nano-TiO_2_ by a negative charge. The negative charges on the nano-TiO_2_ were reduced significantly when ENPs were added to CTAB solution. A significant increase of the positive charge was observed also for nano-ZnO and nano-Ni in CTAB solution. SDBS modified the positive charge of nano-ZnO and nano-Ni, and reduced the negative charge of TiO_2_. Due to the small amounts of surfactants added, pH of the solutions with the surfactants did not differ significantly among the particular variants and was at the range from 6.9 (ISO medium) to 7.3 and a level of O_2_ was above 3 mg/L. Generally, colloidal suspensions with zeta potential above 20 mV and those more negatively charged than −20 mV are considered stable (Prathna et al. 2011). In a majority of the variants, ENPs in the surfactant solutions were characterised by values of zeta potential above 20 mV and bellow −20 mV, which may indicate their stability (Fig. 4). The sole exception was nano-TiO_2_ in TX100 solution, and nano-ZnO in SDBS solution. The presence of TX-100 in the system may cause a shift of the slipping plane, and in consequence an increase of zeta potential. The measurement was made at pH 6.9–7. That is such a level of pH values at which neither ZnO_2_ nor Ni exceed the pHpzc point—for ZnO_2_ it is 9–10 and for Ni also 9–10 (by contrast TiO_2_ has pHpzc between 5 and 6), and therefore in the range studied we have a positive value of the potential, and the shift of the slipping plane can result in its increase. In this case the aggregation of particles in the system may be affected the phenomenon of flocculation, where there is no decrease of the potential as a result of joining of colloids via hydration layers.

#### The toxicity of ENPs to *D. magna*

**Fig. 6:**
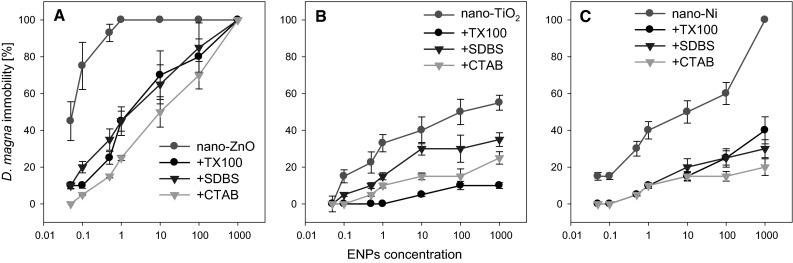
Influence of surfactants on the toxicity of nanoparticles (**a**) nano-ZnO, (**b**) nano-TiO_2_ and (**c**) nano-Ni to *Daphnia magna*. The concentration of surfactants: CTAB and SDBS—0.01 mg/L, TX 100—1 mg/L

The toxicity of the ENPs clearly depended on their kind. Increasing concentration of nano-ZnO, nano-TiO_2_ and nano-Ni caused an increase in the rate of immobility of *D. magna* (Fig. 6). The values of EC_50_ determined for nano-ZnO, nano-TiO_2_ and nano-Ni were 0.031, 99 and 10.3 mg/L, respectively. For nano-ZnO, the values determined were notably lower than those obtained by other authors (Heinlaan et al. 2008; Kahru et al. 2008; Wiench et al. 2009; Blinova et al. 2010). For example, the values of EC_50_ determined by Heinlaan et al. (2008) for *D. magna* were at the level of 3.2 mg/L. Higher values than those observed in this study were noted also by other authors (Wiench et al. 2009; Blinova et al. 2010; Naddafi et al. 2011). Lower values of EC_50_ than those obtained in the studies cited earlier (0.6 mg/L) were obtained for nano-ZnO by Zhu et al. (2010). Nevertheless, those values were still higher by an order of magnitude than those presented in this study. Similarly diversified results were observed by other authors in the case of nano-TiO_2_ (Heinlaan et al. 2008; Kahru et al. 2008; Wiench et al. 2009; Dabrunz et al. 2011; Clément et al. 2013). The literature values of EC_50_ for nano-TiO_2_ indicate from a complete lack of toxicity of nano-TiO_2_ (at the concentration of 10 g/L) to values at the level of as much as 1.3–3 mg/L (Hund-Rinke and Simon 2006). The differences observed in toxicity thresholds among the particular studies may be related to differences in particle size, preparation methods or test designs (Zhu et al. 2010). Whereas, the literature does not provide any information on the subject of toxicity of nano-Ni. Most research is focused on NiO nanoparticles (Gong et al. 2011; Faisal et al. 2013). In a study conducted by Deleebeeck et al. (2008), depending on the properties of the solution, the values of EC_50_ for NiCl_2_ varied from 1.82 to 5.50 mg/L. The high level of EC_50_ observed in this study is probably a result of very low solubility of nano-Ni, which primarily determines the toxicity of ENPs (Kahru et al. 2008).

#### Influence of surfactants on ENPs toxicity to *D. magna*

The addition of the ENPs to solutions of all the surfactants caused a reduction of their toxicity within the whole range of concentrations applied (Fig. 6). The exception were particles of nano-ZnO, in the case of which at the highest concentration tested (1000 mg/L) no significant differences were noted between the toxicity in the solutions with and those without the surfactants (Fig. 6a). The greatest reduction of toxicity for nano-ZnO was observed after the application of CTAB. Depending on the nano-ZnO concentration, the reduction of toxicity of nano-ZnO varied from 30 (100 mg/L) to 100 % (the total reduction of toxicity in the lowest nano-ZnO concentration). The presence of both TX100 and SDBS reduced the toxicity of nano-ZnO at a similar level which varied from 15 to 87 %. The greatest reduction of toxicity (>60 %) was observed at the lowest range of concentrations (0.05–0.5 mg/L nano-ZnO) (Fig. 6a). As in the case of nano-ZnO, all of the surfactants under study also reduced the toxicity of nano-TiO_2_. However, the reduction of toxicity was not as significant as was the case with nano-ZnO. Also, distinct differences were noted in the reduction of toxicity among the surfactants (Fig. 6b). In the case of TiO_2_ the best reduction was obtained for TX100 (80–100 %/the total reduction of toxicity), followed by CTAB (55–100 %/the total reduction of toxicity) and finally SDBS (25–66 %). As opposed to nano-ZnO, greater reduction of toxicity was observed for higher concentrations of nano-TiO_2_. In addition, even at the highest concentration tested the toxicity of nano-TiO_2_ in solutions containing the surfactants was significantly lower compared to the solution without any surfactants. The toxicity of nano-Ni was also reduced under the effect of the surfactants, with no significant differences observed among the surfactants for most of the concentrations tested. The reduction of toxicity (from 0.05 to 10 mg/L nano-Ni) varied from 60 to 100 % (the total reduction of toxicity). Only at the highest concentration tested significant differences were observed among all the surfactants. The best reduction was obtained for TX100 (80 %), followed by SDBS (70 %), and the lowest for CTAB (60 %) for the highest concentration of nano-Ni.

Studies on the effect of surfactants on the toxicity of ENPs are relatively scarce (Barrena et al. 2009; Stampoulis et al. 2009) even though their effect on the behaviour of ENPs in the environment is commonly known (Tkachenko et al. 2006). Stampoulis et al. (2009), similarly to Barrena et al. (2009), observed an increase of the toxicity of ENPs in the presence of surfactants with relation to plants. The results obtained in this study are different, which is most probably related with the fact of using a different test organism.

**Fig. 7:**
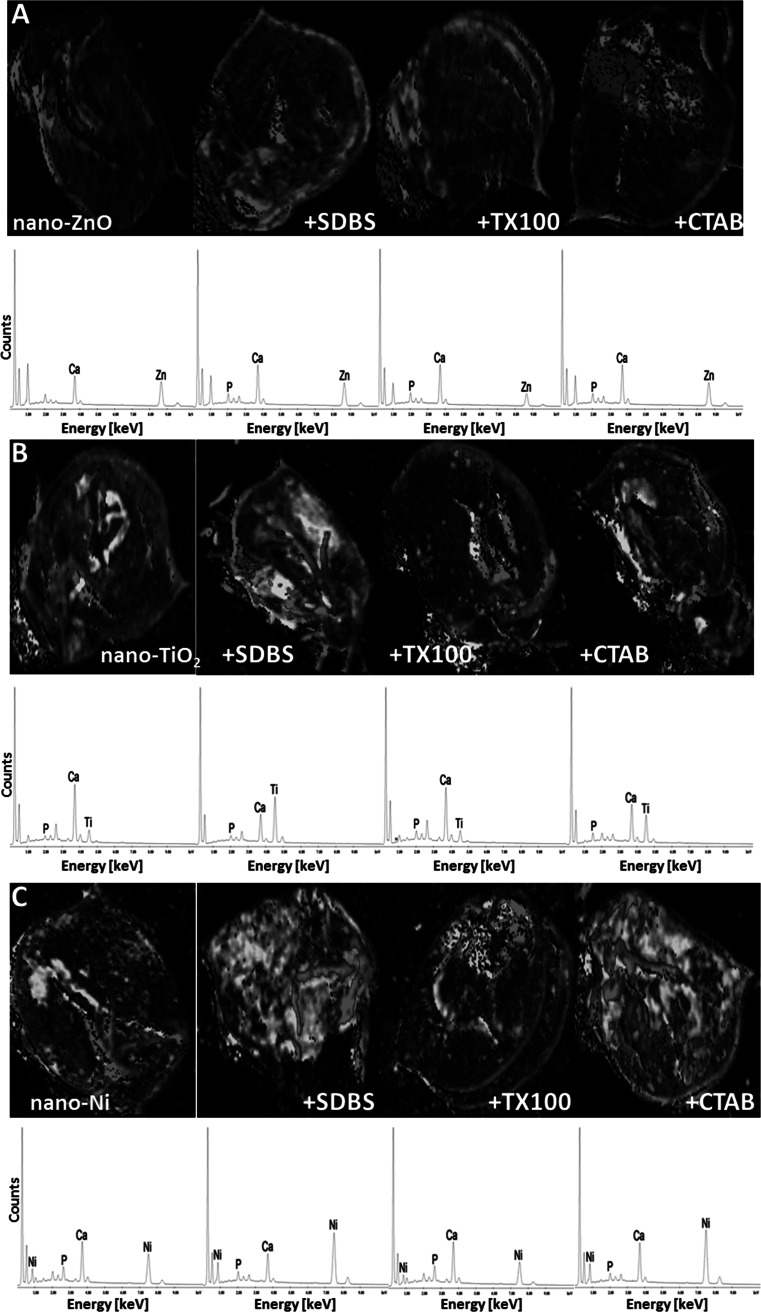
SEM with EDSpictures of *D. magna* exposed to (**a**) nano-ZnO, (**b**) nano-TiO_2_ and (**c**) nano-Ni alone and ENPs in solution of surfactants. The colours of *blue*, *turquoise*, *green* correspond with the presence of Zn, Ti and Ni, respectively (Color figure online)

All of the surfactants used in this study decreased the toxicity of the ENPs tested towards *D. magna* (Fig. 6). At the same time, SDBS and TX100 increased the size of aggregates of all ENPs tested at the beginning of the experiment, while CTAB only after 24–48 h. This may indicate that the formation of aggregates/complexes of ENPs with surfactants (Fig. 2, 3, 4) inhibits the accumulation of ENPs by *D. magna* or reduces surface coating and, indirectly, also molting inhibition, which may result in loss of mobility (Dabrunz et al. 2011; Kwon et al. 2015). At the start of the experiment, aggregates of ENPs without the surfactants were characterised by particle size >50 μm, and the addition of the surfactants increased the mean size of the aggregates to above 80 μm (Fig. 2). Hund-Rinke and Simon (2006) considered that particles with a diamter of less than 50 μm are ingested by *D. magna* without any selective mechanism. However, larger particles are too big, and *D. magna* prevent them from entering the filter chamber or reject them through movement of the postabdominal claw (Clément et al. 2013). Kwon et al. (2015) also observed that uptake of NPs into *D. magna* are strongly dependent on their aggregation (i.e., hydrodynamic sizes), rather than their core sizes. This finds support in SEM–EDS images (Fig. 7). Unfortunately, based on the images acquired we cannot identify whether the ENPs are on the surface or inside *D. magna*. In the Fig. 7 we can clearly see lower concentration of Zn on the surface/inside *D. magna* in solution with SDBS and TX100 than in the solutions with the ENPs alone (Fig. 7a). Although at the start of the experiment nano-ZnO in CTAB solution was characterised by small size of aggregates, after 24–48 h their significant increase was also observed (Fig. 4). Thus at the beginning the availability of nano-ZnO in CTAB solution was probably higher than in other surfactant solutions. This explains the higher concentration of Zn (Fig. 7a) in the nano-ZnO-CTAB solution compared to solutions with SDBS and TX100. However, the concentration of Zn is still lower relative to nano-ZnO alone, which in turn explains the reduction of toxicity.

Although a reduction of toxicity after the addition of the surfactants was observed for all ENPs, the hypothesis suggested earlier that the reduction of bioavailability or surface coating reduces the toxicity of ENPs in the presence of surfactants cannot be applied to nano-TiO_2_ and nano-Ni. Both for nano-TiO_2_ (Fig. 7b) and nano-Ni (Fig. 7c) the concentration of ENPs on the surface/inside of *D. magna* was distinctly higher than or similar to that in solutions containing nano-TiO_2_ in SDBS solution and nano-TiO_2_ in CTAB solution as well as nano-Ni in SDBS solution and nano-Ni in CTAB solution compared to experiment without surfactants. In spite of that, the surfactants reduced the toxic effect of the ENPs. However only in the case of nano-TiO_2_ clear regularity may be observed. One can clearly see that the reduction of toxicity (Fig. 6b) in the presence of surfactants depends on the concentration of Ti on the surface/inside of *D. magna* (Fig. 7b). No similar relation was noted in the case of nano-Ni (Fig. 7c). Analysing the results relating to nano-TiO_2_ and nano-Ni one should assume that the reduction of toxicity of ENPs in the presence of surfactants may have another/additional mechanism than just accumulation/surface coating of ENPs. Gaiser et al. (2011) suggested that besides particle size and solubility, interactions between particles and food materials in the test media may affect the toxicity of ENPs. However, during the test (*D. magna* was only feed before experiment according to the procedure), which excludes that factor as a possible one. Das et al. (2013) suggest that the toxicity of ENPs is a combination of the release of ions from particles and *D. magna* direct interactions with the ENPs. Reduction of toxicity undoubtedly results from interactions of ENPs with surfactant particles. Dabrunz et al. (2011) demonstrated that after a few hours most of nano-TiO_2_ sink to the bottom of the test beakers. The addition of surfactants to the solution increases the size of aggregates, as a result of which they become heavier and sediment faster compared to ENPs alone (Chibowski et al. 2007). ENPs settled on the substrate are not only harder to uptake by *D. magna* than when suspended in the solution, but also direct contact of ENPs with *D. magna* is limited. In addition, the binding of ENPs by surfactants may inhibit the solubility of ZnO and Ni, which—according to certain authors—determines primarily the ecotoxicity of ENPs. That last issue, however, requires additional research.

## Conclusions

The presence of surfactants considerably reduced the toxicity of all tested ENPs. Although earlier studies showed that surfactants may increase the toxicity of ENPs towards plants, an opposite tendency was observed in this study. This indicates that in the analysis of the toxicity of ENPs it is very important to take into account various groups of organisms, because of different potential mechanisms of ENPs toxicity. Generalization of results may lead to erroneous conclusions. A positive aspect of the results obtained is that the toxicity of ENPs can be reduced as a result of their contact with surfactants, which reduces the risk to the environment. On the other hand, however, surfactants—increasing the aggregation of ENPs—reduce their mobility and that may mean a longer time of retention of those contaminants. These problems assume a growing importance in view of the every greater production of surfactants and ENPs alone. It should also be emphasised that surfactants are used for the stabilisation of ENPs in ecotoxicological studies. That may lead to incorrect estimation of the toxicity of ENPs. Underestimation of the environmental hazard may lead to serious environmental consequences, with potential effect on various groups of organisms.
